# Evaluation of an Optimal Cut-Off Point for the Ki-67 Index as a Prognostic Factor in Primary Breast Cancer: A Retrospective Study

**DOI:** 10.1371/journal.pone.0119565

**Published:** 2015-07-15

**Authors:** Rumiko Tashima, Reiki Nishimura, Tomofumi Osako, Yasuyuki Nishiyama, Yasuhiro Okumura, Masahiro Nakano, Mamiko Fujisue, Yasuo Toyozumi, Nobuyuki Arima

**Affiliations:** 1 Surgery, Kumamoto City Hospital, Kumamoto, Japan; 2 Breast and Endocrine Surgery, Kumamoto City Hospital, Kumamoto, Japan; 3 Pathology, Kumamoto City Hospital, Kumamoto, Japan; Queen Mary Hospital, HONG KONG

## Abstract

The Ki-67 index is an important biomarker for indicating the proliferation of cancer cells and is considered to be an effective prognostic factor for breast cancer. However, a standard cut-off point for the Ki-67 index has not yet been established. Therefore, the aim of this retrospective study was to determine an optimal cut-off point in order to establish it as a more accurate prognostic factor. Immunohistochemical analysis of the Ki-67 index was performed on 4329 patients with primary breast cancer from August 1987 to March 2012. Out of this sample, there were 3186 consecutive cases from September 1997 with simultaneous evaluations of ER, PgR and HER2 status. Cox's proportional hazard model was used to perform univariate and multivariate analyses of the factors related to OS. The hazard ratios (HR) and the p values were then compared to determine the optimal cut-off point for the Ki-67 index. The median Ki-67 index value was 20.5% (mean value 26.2%). The univariate analysis revealed that there was a statistically significant negative correlation with DFS and OS and the multivariate analysis revealed that the Ki-67 index value was a significant factor for DFS and OS. The top seven cut-off points were then carefully chosen based on the results of the univariate analysis using the lowest p-values and the highest HR as the main selection criteria. The multivariate analysis of the factors for OS showed that the cut-off point of 20% had the highest HR in all of the cases. However, the cutoff point of 20% was only a significant factor for OS in the Luminal/HER2- subtype. There was no correlation between the Ki-67 index value and OS in any of the other subtypes. These data indicate that the optimal cut-off point of 20% is the most effective prognostic factor for Luminal/HER2- breast cancer.

## Introduction

Multi-gene assays, primarily derived from proliferation genes [1], provide valuable prognostic information for medical professionals and cancer patients. In fact, cell proliferation is one of the most important prognostic factors for patients with aggressive tumors and for overall patient survival [2, 3]. Within cell proliferation the biomarkers in particular have proven to be effective in the formulation of a more accurate prognosis and therefore further research in this area is warranted. Out of all the biomarkers associated with cell proliferation, Ki-67 is the most suitable candidate for breast cancer research because it is expressed in almost all normal and malignant cells and it is an easy and reliable method of assessing the cell cycle pathways. Moreover, the prognostic significance of this biomarker in breast cancer research has been well documented [2–5]. Studies have shown that high Ki-67 index values are associated with tumors that are categorically graded high, are large, have positive lymph node involvement, and belong to either the triple negative or HER-2 positive subtypes [6, 7]. Other studies have found that the Ki-67 index is a key determinant to predict tumor response to adjuvant systemic treatments such as chemotherapy [8] and aromatase inhibitors [9]. The 2011 St. Gallen’s International Expert Consensus found that the Ki-67 index is effective in discriminating between Luminal A and B type tumors and recommended a cut-off point of 14% [10]. This was the first time that any attempt was made at trying to establish a standard international cut-off point for the Ki-67 index as a prognostic factor for breast cancer [11].

The guidelines of the American Society of Clinical Oncology (ASCO) do not include Ki-67 as one of the required biological markers to routinely investigate when examining breast cancer patients [12] despite the growing body of evidence showing that it is a clinically useful and widely accepted biomarker. Neglecting to establish a standard cut-off point for the Ki-67 index has resulted in global inconsistencies in scoring methodology and has prevented direct comparisons of Ki-67 values across laboratories and clinical trials. Moreover, improper handling and preparation of tissue samples tend to generate false Ki-67 index values which could possibly lead to erroneous conclusions about the biology of the tumor. This usually happens in cases where there was a delayed time to fixation, longer fixation time, and/or insufficient conditions of fixation. Thus, the pre-analytical setting critically influences the Ki-67 labeling and the care in handling the pre-analytical tissue is crucial in determining accurate Ki-67 values [13]. At our institute, the Ki-67 index was evaluated using the same procedure for every patient with breast cancer for the past 20 years. [7] The aim of this retrospective study was to establish the optimal cut-off point for the Ki-67 biomarker so that it can be an effective prognostic factor for disease-free survival (DFS) and overall survival (OS) in primary breast cancer cases. This study was approved by the ethics committee at Kumamoto City Hospital, Japan.

## Patients and Methods

### Patients

This study examined 4329 consecutive primary breast cancer (stage I~III) cases from August 1987 to March 2012 at Kumamoto City Hospital, Japan. Out of these patients (Table 1), 3186 consecutive cases from September 1997 were simultaneously evaluated as having estrogen receptor (ER), progesterone receptor (PgR) and HER2 status. The Ki-67 index values were also calculated for each cases to assist with prognosis. Informed consent was obtained from all of the patients. The age of the patients ranged from 23 to 95 years (mean 55.8) and the mean tumor diameter was 2.2 cm (range 0.1–34). Two-thirds (64.6%) of the patients had pathologically negative nodes. The ER- and PgR-positive rates were 66.4 and 41.8%, respectively. The positive rate of HER2 cases was 9.8% and the positive rate of p53 overexpression was 17.2% (Table 1). The distribution of patients according to breast cancer subtypes were as follows; Luminal/ HER2-type (2426 cases), Luminal/HER2+ type (282 cases), HER2 enriched type (178 cases), and triple negative type (300 cases). Patient characteristics are available for inspection in Table 2. The median observation period was 81 months.

**Table 1:** Patients Characteristics in Primary Breast Cancer.

Variables		All cases
No. of cases		4329
Age (mean / median)		55.8 / 54
Ki-67 (mean / median)		26.2/ 20.5
Menopausal status	pre	1801(41.6%)
	post	2520(58.2%)
	unknown	8(0.2%)
Nuclear grade	1or 2	3185(73.6%)
	3	631(14.6%)
	unknown	513(11.8%)
Tumor size (mm)	<20	2417 (55.8%)
	≥20	1754 (40.5%)
	unknown	158(3.7%)
p53 expression	<50%	2828 (5.3%)
	≥50%	728 (16.8%)
	unknown	773(17.9%)
Lymph node metastasis	-	2795 (64.6%)
	+	1454 (33.6%)
	unknown	80(1.8%)
ER	<1%	497 (11.5%)
	≥1%	3832(88.5%)
PgR	<1%	757 (17.5%)
	≥1%	2632(60.8%)
	unknown	940(21.7%)
Surgical method	Total mastectomy	2018 (46.6%)
	Partial mastectomy	2259 (52.2%)
	unknown	52 (1.2%)
Adjuvant therapy	Endocrine therapy alone	1907 (44.1%)
	Chemotherapy alone	636 (14.7%)
	Chemo-endocrine therapy	1123 (25.9%)
	Trastuzumab therapy	104 (2.4%)
	none	525(12.1%)
	unknown	34 (0.8%)

**Table 2:** Patients Characteristics according to Subtypes.

Variables		Luminal /HER2 -	Luminal /HER2+	HER2 enriched	Triple negative	P-value
No. of cases		2426	282	178	300	
Age (mean / median)		56.6 / 55	53.3 / 53	57.3 / 57	58.5 / 58	
Ki-67(%) (mean / median)		23.2 / 19	35.5 / 35	43.0/ 41	51.9 / 51	<0.001
Menopausal status	pre	987	122	48	83	
	post	1432	160	130	217	
	unknown	7	-	-	-	
Nuclear grade	1or 2	2170	195	72	123	<0.001
	3	201	82	104	171	
	unknown	106	5	2	6	
Tumor size	<20 mm	1401	127	98	148	<0.001
	≥20mm	919	136	69	140	
	unknown	106	19	11	12	
p53 expression	<50%	2139	162	80	145	<0.001
	≥50%	285	116	98	155	
	unknown	2	4	-	-	
Lymph node metastasis	-	1581	175	123	192	<0.001
	+	794	102	50	101	
	unknown	51	5	5	7	
ER	<1%	11	8	178	300	
	≥1%	2415	274	-	-	
PgR	<1%	215	61	178	300	
	≥1%	2053	146	-	-	
	unknown	158	75	-	-	
Surgical operation	Total mastectomy	909	136	87	116	
	Partial mastectomy	1479	141	88	179	
	unknown	38	5	3	5	
Adjuvant therapy	Endocrine therapy alone	1510	44	0	4	
	Chemotherapy alone	47	27	76	190	
	Chemo-endocrine therapy	624	134	1	5	
	Trastuzumab therapy	-	47	50	-	
	none	226	30	51	100	
	unknown	19	-	-	1	

### Histopathological Examination

The factors investigated included the presence or absence of lymph node metastasis, nuclear grade, ER/PgR status, Ki-67 index value, HER2 and p53 overexpression. Immunostaining for ER, PgR, p53, Ki-67 and HER2 was done following the same procedure described by Kai et al. (2006) in the International Journal of Clinical Oncology [14]. The positive cell rates for ER/PgR were determined by immunohistochemistry (IHC) and a value of ≥1% was considered positive. The proliferative activity was determined by immunostaining using the Ki-67 antibody (clone MIB-1; Dako, Glostrup, Denmark) and an autostainer (Ventana, Tucson, USA). The staining was evaluated by two experienced pathologists. The fraction of proliferating cells (positive for Ki-67) was calculated based on a count of at least 500 tumor cells in the relatively dense concentration of positive cancer nuclei, hereafter known as the “hot spot”. Percentages of positive cells were calculated and used as the Ki-67 index. The p53 and HER2 expressions were evaluated by immunostaining (LSAB method) with the mouse monoclonal anti-p53 antibody (clone DO7; Dako) and the Hercep Test (Dako). The status of p53-positive cells ≥50% was classified as p53 overexpression [15]. The staining pattern of HER2 was divided into the following four groups: 3+ (strong and diffuse staining), 2+ (moderate and diffuse staining), 1+ (focal staining >10% cancer cells) and 0 (negative).

### Breast Cancer Subtypes

Breast cancer is classified by gene expression profile into subtypes. IHC surrogate panels have also been proposed to potentially identify the molecular-based groups [10, 11]. In this study, ER and/or PgR positivity and HER2 negativity were classified as Luminal/HER2–; ER and/or PgR positive and HER2+ as Luminal/HER2+; ER and PgR negative and HER2 positive as HER2 enriched; and ER, PgR negative and HER2 negative as triple negative (TN). HER2-positive tumors were defined in cases with HER2 IHC as 3+ or 2+ and a FISH amplification ratio of >2.0.

### Adjuvant therapy

Postoperative adjuvant therapy has been performed since 1999 based on the recommendations made at the St. Gallen’s International Meeting (1998). The chemotherapy regimen for patients before 1999 was oral fluorouracil agents, CMF (cyclophosphamide/methotrexate/fluorouracil) combination and anthracycline, and the chemotherapy regimen for patients after 1999 were anthracycline and taxanes. Trastuzumab was added as an adjuvant treatment in 2008. Postoperative follow-up examinations were performed every 3 months until 3 years after surgery, every 3–6 months until 3–5 years after surgery, and every 6–12 months until 5–10 years after surgery. Patients underwent chest X-ray, mammography, tumor marker tests and abdominal ultrasonography once a year and CT scans were performed after consultation with the patients. From the 10th year, patients underwent mammography and were asked to visit the hospital if they had any concerns.

### Statistical Analysis

The intergroup comparisons (Table 2) were done using the chi-square test and Fisher’s exact test. Age and the mean tumor diameter were determined using Wilcoxon’s nonparametric test. Cumulative DFS and OS were calculated using the Kaplan-Meier method and tested using the log-rank test. Uni- and multivariate analyses for factors related to recurrence were performed using the Cox proportional hazard model (SPSS version 21). To determine whether the sample size was appropriate for analysis, each model was evaluated using the Harrell C-index which is a continuous index that ranges from 0 to 1.0 and the higher the value the better the model [16, 17]. For the overall sample size, the C-index was 0.74 for all the patients and 0.72 for the patients with luminal tumors. This analysis revealed that the overall sample size and the sample size for each of the subgroups were appropriate for this study.

## Results

### Disease-free and overall survival and the Ki-67 index value as continuous variables

A multivariate analysis was conducted using the Ki-67 index values as continuous variables to investigate the prognostic significance of this index in primary breast cancer. The multivariate analysis revealed that the Ki-67 index value, tumor size, p53 overexpression and lymph node status were significant factors (Table 3).

**Table 3:** Multivariate Analysis of Factors for Disease-free and Overall Survival in Primary Breast cancer.

		Diseases—free survival		Overall survival	
	Category	P value	Hazard ratio (95%CI)	P value	Hazard ratio (95%CI)
Nuclear Grade	3/2,1	0.006	1.546 (1.136–2.106)	0.13	1.299 (0.926–1.821)
Tumor size	≥20/<20mm	<0.001	2.382 (1.856–3.056)	<0.001	2.236 (1.694–2.951)
Ki-67 index	continuous	<0.001	1.014 (1.008–1.020)	<0.001	1.014 (1.008–1.020)
p53over- expression	+/-	<0.001	1.741 (1.347–2.249)	0.001	1.612 (1.215–2.140)
Node Status	+/-	<0.001	6.265 (4.626–8.484)	<0.001	4.377 (3.310–6.328)

### Ki-67 index values and subtypes

The distribution of cases were as follows: 2426 Luminal/HER2- cases (76.1%); 282 Luminal/HER2+ cases (8.9%); 178 HER2 enriched cases (5.6%) and 300 TN cases (9.4%). The mean/median Ki-67 values for all the patients were 26.2% and 20.5%, respectively. The mean/median Luminal/HER2- values were 23.2% and 19%, the Luminal/HER2+ values were 35.5% and 35%, the HER2 enriched values were 43.0% and 41% and the TN tumor values were 51.9% and 51%, respectively. There was a significant difference in these values and a significant difference among the subtypes (Table 2).

### Univariate analysis for DFS and OS using different Ki-67 cut-off points

Univariate analysis for DFS and OS was performed on the data for all the patients using different Ki-67 cut-off points (Table 4). There were significant relationships between several Ki-67 cut-off points (every 5% from 10% to 60%) and DFS/OS. The top seven cut-off points were selected from the univariate analysis according to lower p-values and higher Hazard ratios and then a multivariate analysis for OS was conducted.

**Table 4:** Univariate analysis for Disease-Free and Overall Survival using Different Ki-67 cut-off points.

	Diseases—free survival		Overall survival	
Cut-off point	P value	Hazard ratio (95%CI)	P value	Hazard ratio (95%CI)
10%	<0.001	1.846 (1.451–2.347)	<0.001	1.683 (1.310–2.162)
15%	<0.001	2.067 (1.713–2.493)	<0.001	1.855 (1.525–2.257)
20%	<0.001	2.274 (1.933–2.675)	<0.001	2.287 (1.919–2.726)
25%	<0.001	2.146 (1.841–2.502)	<0.001	2.211 (1.876–2.610)
30%	<0.001	2.137(1.835–2.489)	<0.001	2.214(1.876–2.612)
35%	<0.001	2.190 (1.876–2.558)	<0.001	2.350 (1.987–2.779)
40%	<0.001	2.282 (1.946–2.676)	<0.001	2.383 (2.006–2.832)
45%	<0.001	2.247 (1.901–2.655)	<0.001	2.410 (2.015–2.883)
50%	<0.001	2.230 (1.871–2.658)	<0.001	2.436 (2.022–2.935)
55%	<0.001	1.937 (1.583–2.370)	<0.001	1.971 (1.586–2.450)
60%	<0.001	1.700 (1.340–2.158)	<0.001	1.793 (1.387–2.319)

### Multivariate analysis of Factors for OS according to the Ki-67 cut-off points

We conducted a multivariate analysis of factors for OS using the top seven Ki-67 cut-off points with the lowest p-value and highest HR and the results revealed that the optimal cut-off point was 20% (Table 5).

**Table 5:** Multivariate analysis of Factors for Overall Survival according to the Selected Ki-67 cut-off points.

	Overall survival	
Cut-off point	P value	Hazard ratio (95%CI)
20%	<0.001	1.880 (1.372–2.575)
25%	<0.001	1.838 (1.380–2.448)
30%	0.001	1.618 (1.231–2.127)
35%	<0.001	1.694 (1.296–2.215)
40%	<0.001	1.713 (1.308–2.243)
45%	<0.001	1.712 (1.302–2.282)
50%	<0.001	1.617 (1.207–2.166)

### Survival based on the Ki-67 value according to breast cancer subtypes

**Fig 1:**
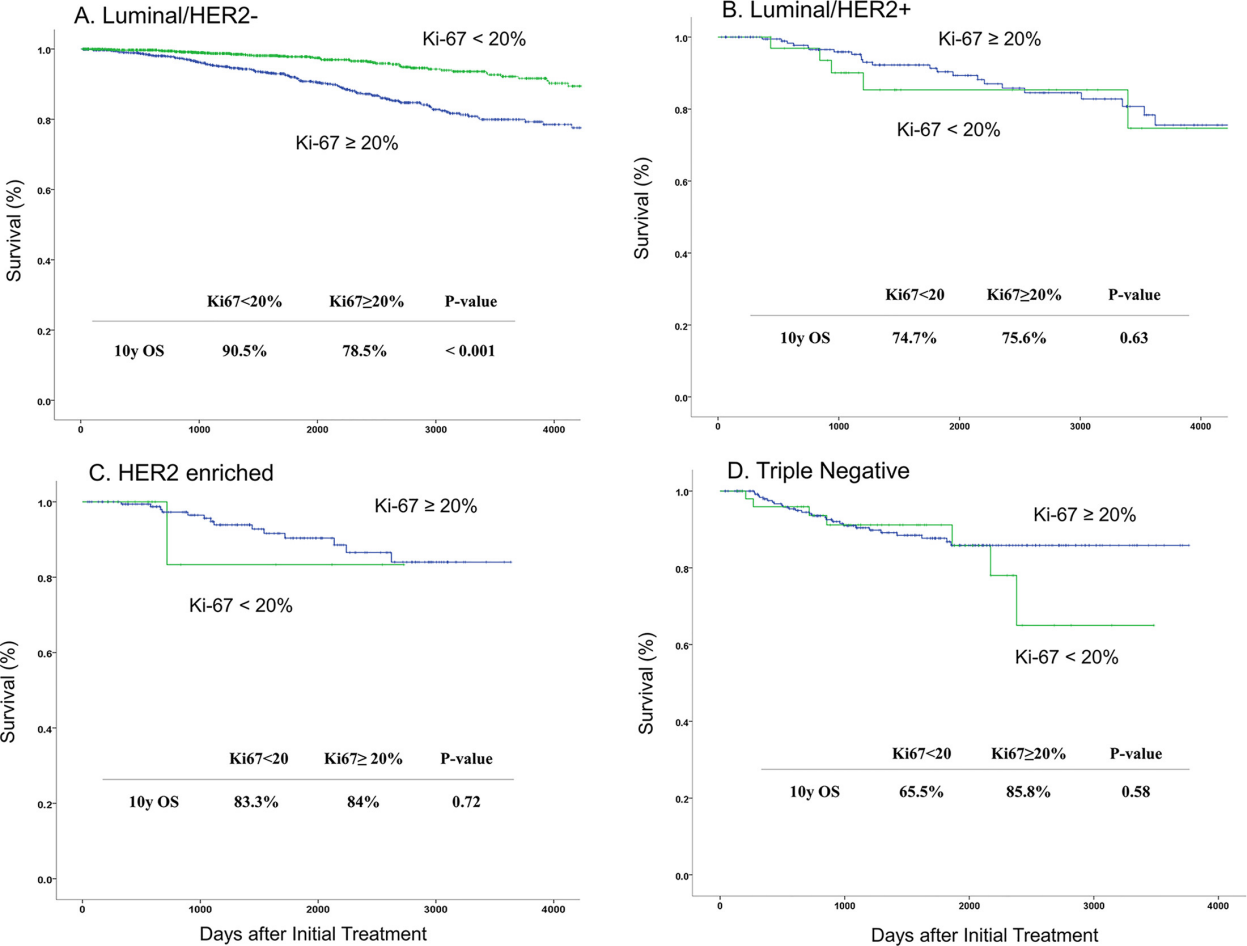
Overall survival based on the Ki-67 cut-off point of 20% according to breast cancer subtypes. (The OS rates of patients with lower Ki-67 values (<20%) was significantly higher than those with higher Ki-67 values (≥20%) in the luminal/HER2- subtype (A). However, there were no significant correlations between the Ki-67 cut-off point of 20% and survival in any of the other subtypes (B, C, D).)

The cumulative OS rates in patients with Luminal/HER2- type tumors were calculated using a Ki-67 cut-off point of 20%. The OS rates of patients with lower Ki-67 values (<20%) was significantly higher than those with higher Ki-67 values (≥20%). However, there were no significant correlations between the Ki-67 cut-off point of 20% and survival in any of the other subtypes (Fig 1).

## Discussion

The main focus of this retrospective study was to identify the optimal cut-off point for the Ki-67 index so that it could be used as an important prognostic factor for primary breast cancer. Our analysis revealed that a wide range of cut-off points was significant for DFS and OS in all the patients. The median Ki-67 value was 20.5% (mean value 26.0%) in the hot spot of 4329 consecutive cases of primary breast cancer. The multivariate analysis for OS indicated that the optimal cut-off point was 20% in all of the cases. Higher Ki-67 values (≥20%) in all of the cases and Luminal/HER2- type breast cancer significantly correlated with lower DFS and OS rates.

The St. Gallen’s International Expert Consensus recommended endocrine therapy for the ER-positive cases and anti-HER2 therapy for the HER2-positive cases and to use the Ki-67 index as an effective tool to distinguish between Luminal A and B type tumors [10]. Moreover, the St. Gallen’s International Expert Consensus recommended a cut-off point of 14% for the Ki-67 labeling index [18]. However, the cut-off point between ‘high’ and ‘low’ values for the Ki-67 index still varies between laboratories [11, 19]. We found that the Ki-67 index value was a significant factor for DFS and OS using several cut-off points (including 15% and 20%). The multivariate analysis of the factors for OS showed that the cut-off point of 20% had the highest HR in all of the cases.

The univariate analysis of various Ki-67 cut-off points for OS (Table 4) revealed that there was a significant difference in each of the cut-off points. The univariate analysis also revealed that the HR (2.5) for the Ki-67 index was the highest at 50%, indicating that perhaps there are more than one optimal cut-off point. Therefore, we divided the Ki-67 index values into 3 groups using a cut-off point of 20% and 50%. The median value of the Ki-67 index was 50% in the TN tumors because they have a higher risk of early recurrence after initial surgery [20]. Most of the patients with a Ki-67 value of ≥50% did not have late recurrence. On the other hand, there were more cases with pCR when the Ki-67 index values were higher. In our previous study, the mean Ki-67 value of tumors with pCR was 63.3% (>50%) and that with non-pCR was 45.0% (<50%). We also found that there was a significant difference between the two groups (p = 0.002). There was no pathological responder in the cases with a Ki-67 index value <25% [21]. Some studies reported a similar tendency with a mean Ki-67 value of 50.6 ± 23.4% in patients with pCR, and patients without pCR had an average of 26.7 ± 22.9% positively stained cancer cells [22]. Furthermore, the Ki-67 index values were significantly elevated after recurrence and the cases with a Ki-67 index value of ≥50% significantly increased [23]. These findings suggest that to divide the patients into 3 groups according to the Ki-67 index using the cutoff points of 20% and 50% is clinically meaningful. The Ki-67 index is a consecutive variable. For the purpose of this study, the cut-off values of 20% and 50% have great clinical meaning because 50% percent represents a high proliferative potential in TN tumors and 20% is the median of all the cases. The neoadjuvant GeparTrio trial [24] used the following cut-off points for a detailed evaluation: Ki-67 low (≤15%), Ki-67 intermediate (15.1%–35%), and Ki-67 high (>35%). The univariate and multivariate analyses revealed significant pCR, DFS and OS rates for the three groups. This means that the cut-off point may vary depending on the purpose, subtype and subjects of the study.

Gong et al. investigated the correlation between the location of the tumor and prognosis, and found that the Ki-67 distribution pattern (negative, diffuse type and borderline type) was an independent prognostic factor [25]. In this study, the hot spot was the main area evaluated. Although calculating an overall average score was recommended by the International Ki-67 Breast Cancer Working Group [26], several studies [27, 28] reported that the Ki-67 index value at the hot spot was significantly correlated with survival. The Working Group also stated that cut-off points for prognosis, prediction, and monitoring should only be applied if the results from the local practice have been validated against those in studies that have defined the cut-off values for the intended use of the Ki-67 result [13]. Limitations of this study include the retrospective nature of the analysis and the study population was not homogeneous in terms of the drugs used as adjuvant regimens. The findings suggest that tumor biology strongly influences treatment efficacy and prognosis. Although Oncotype Dx, uPA and PAI-1 were not included in this study, it can be argued that the Ki‑67 index may support therapeutic decisions in cases where these factors are not feasible.

## Conclusion

We calculated the Ki-67 index value in the hot spot of 4329 consecutive cases of primary breast cancer and found that on a distribution ranging from <1% to 99% the median value was 20.5%. In other words, the optimal cut-off point for the Ki-67 index as an effective prognostic factor for OS was 20% because it had the highest HR value and the lowest p value. However, the Ki-67 value was only a significant prognostic factor for the Luminal/HER2- subtype. Therefore, standardization of Ki-67 assessment is important in order to use this biomarker in clinical practice.
